# Drug Release from Nanoparticles (Polymeric Nanocapsules and Liposomes) Mimed through a Multifractal Tunnelling-Type Effect

**DOI:** 10.3390/polym15041018

**Published:** 2023-02-17

**Authors:** Elena Simona Băcăiță, Delia Mihaela Rață, Anca Niculina Cadinoiu, Vlad Ghizdovăț, Maricel Agop, Alina-Costina Luca

**Affiliations:** 1 Faculty of Machine Manufacturing and Industrial Management, Gheorghe Asachi Technical University of Iasi, D. Mangeron Bld. No. 73, 700050 Iasi, Romania; 2 Faculty of Medical Dentistry, “Apollonia” University of Iasi, Muzicii Street No. 2, 700511 Iasi, Romania; 3 Department of Biophysics and Medical Physics, “Grigore T. Popa” University of Medicine and Pharmacy, 700115 Iasi, Romania; 4 Academy of Romanian Scientists, Splaiul Independentei Street No. 54, 050094 Bucharest, Romania; 5 Department of Mother and Child Medicine-Pediatrics, “Grigore T. Popa” University of Medicine and Pharmacy, 700115 Iasi, Romania

**Keywords:** drug release, nanocapsules, liposomes, multifractal tunnelling-type effect

## Abstract

The present study analyzes (theoretically and experimentally) a drug release process from nanoparticles (polymeric nanocapsules and liposomes). This process is functionalized on the surface with an aptamer. These types of drug release processes can also be included in cream-type formulations. The obtained cream ensures the active targeting of tumor epithelial cells, in the case of skin cancer, because it can be easily administered to the skin by spreading, thus avoiding side effects caused by the toxicity of the drug to healthy cells, increasing both patient compliance and the effectiveness of the treatment. The process of obtaining these formulations is a simple one, easy to use and highly reproductible. The theoretical model, based on the multifractal tunnel effect within the Scale Relativity Theory, considers the system as a complex one. In this model, complexity is replaced with system multifractality, quantified in physical quantities as multifractal dimensions and multifractal functions. The main advantage of this approach consists in the fact that it allows us to obtain information on system behavior at a microscopic level and to evaluate microscopic characteristics of the system, such as intrinsic transparences of the drug molecules, multifractal constants as indicators of the system’s complexity, the frequency of interactions within the system and the energy ratio between potential barrier energy and the energy of drug molecules.

## 1. Introduction

Nanomedicine, a branch of medicine, represents a field that has continuously developed and one that uses the knowledge of nanoscience in the prevention, diagnosis and treatment of various diseases and different types of nanocarriers to increase therapeutic effectiveness and reduce the adverse effects associated with conventional treatments [1,2]. For the treatment of cancer, many drug-loaded systems have been studied in the literature, such as micelles [3], liposomes [4,5], polymeric and solid lipid nanoparticles as well as inorganic nanoparticles [6,7,8]. With the continuous development of nanotechnology, polymeric nanocapsules (defined as vesicular systems formed by a hollow core and a polymeric shell with a hydrogel character) have received great interest as a new drug delivery system due to their major advantages such as the improvement of absorption, metabolism and elimination of incorporated drugs and the ability to protect the drugs and avoid their physical–chemical degradation, reducing drugs’ hepatotoxicity [9,10]. The most important characteristics of polymeric nanocapsules are increased stability in contact with biological fluids, low-dimensional polydispersity, spherical shape, high encapsulation capacity of active principles and the ability to release drugs in a prolonged and controlled manner [11]—increasing the degree of intracellular penetration and, consequently, decreasing adverse effects and increasing patient compliance. These advantages make them ideal candidates for the encapsulation of antitumoral drugs [12,13].

A wide range of polysaccharides (sodium alginate, chitosan, starch), proteins (albumin, gelatin, collagen), polyacetates, polylactides and polyanhydrides have been used for obtaining polymeric nanoparticles [14]. Chemical, physical and biological characteristics of the selected polymer are very important for the preparation of the nanoparticles and many factors must be considered prior to preparation, i.e., increased purity, low toxicity, good reproducibility, ease of their manufacture on a larger scale, compatibility with the drug molecule, degradation kinetics adapted to correspond to various types of applications and the flexibility to produce various release profiles [15,16].

On the other hand, liposomes are successful nanocarrier platforms for controlled, prolonged, targeted drug release [17]. Liposomes are self-assembled spherical systems, composed of lipidic bilayers, which incorporate part of the hydrophilic solvent in the inner core [18]; their advantage lies in the fact that they are biocompatible and can encapsulate hydrophilic active principles in the aqueous core and lipophilic active principles within the double-layered lipidic membrane [19,20,21,22]. It is known that liposomes are able to retain lipophilic drugs into their lipidic bilayer, but these lipophilic drugs are not encapsulated with high efficiency without disturbing the integrity of the membrane bilayer.

Nanoparticles functionalized with ligands and loaded with anticancer drugs were proven to have the ability to target tumor tissue and to efficiently release the loaded drug [23]. To target the antitumor drugs directly to the tumor, nanoparticles were functionalized with specific ligands that interact with the receptors overexpressed by cancer cells [24,25,26,27,28,29]. Nanoparticles’ functionalization with aptamers demonstrated that can be recognized much more easily by tumor cell receptors. Additionally, unlike antibodies, the aptamer molecule is smaller, has a lower production cost and shows better stability [30,31]. This study aimed to obtain cream formulations based on nanocapsules/liposomes containing an antitumoral drug (5-fluorouracil) and functionalized with aptamer type AS1411, which may have applications in skin cancer therapy [32]. The AS1411 aptamer is a ligand that targets the nucleolin of tumor cells and inhibits their growth [33,34,35,36]. In our previous articles [17,37], we demonstrated the benefits of nanocapsules and liposomes as platforms for drug release, as well as their ability to bind ligands to their surface in order to target tumor cells. Consequently, a topical formulation (cream) was previously obtained and the drug-loaded nanocapsules and liposomes were designed and characterized and then incorporated into the cream formulation to demonstrate their potential use in the treatment of skin cancer. Aptamers are very versatile multifunctional molecules and have been successfully used for the functionalization of different types of nanoparticles [38].

## 2. Experimental Results

### 2.1. Materials

Some of the materials were purchased from Sigma-Aldrich (St. Louis, MI, USA): (chitosan (CS) with 75–85% degree of deacetylation, dimethylsulfoxide (DMSO), acetone, hexane, surfactants (Tween 80, Span 80), 5-fluorouracil, 1-ethyl-3- (3-dimethylaminopropyl) carbodiimide (EDAC), N-hydroxysuccinimide (NHS), Strat-M^®^ membrane (transdermal diffusion test model) 25mm, PEG 400 and Pluronic F-108. Tris(2-carboxyethyl)phosphine hydrochloride (TCEP•HCl), cholesterol (CHOL), and 5-fluorouracil (5-FU) were procured from Alfa Aeser (Ward Hill, MA, USA). DSPE–PEG–maleimide (DSPE–PEG–MAL) was purchased from Iris Biotech GmbH (Marktredwitz, Germany) and Lipoid E PC S (egg yolk phosphatidylcholine content: ≥96%) (PC) was received as a gift sample from Phospholipid GmbH (Köln, Germany). The DNA aptamer (AS1411-SH) was purchased from Integrated DNA Technologies, Inc. (Coralville, IA, USA). Chloroform and Triton X were obtained from VWR Chemicals (Radnor, PA, USA). Almond oil, Monoi de Tahiti oil, Olliva emulsifying agent, plant-based collagen and Cosgard were acquired from Elemental SRL, (Rome, Italy). Poly (N-vinylpyrrolidone-alt-itaconic anhydride)–poly (NVPAI) with molecular weight value of 100.000 g/mol was synthesized and characterized in our laboratory by a radical copolymerization method [13]. Aptamer-functionalized chitosan (CA) was obtained in the laboratory by an esterification reaction (with N-hydroxysuccinimide and 1-ethyl-3-(3-dimethylaminopropyl)carbodiimide), succeeded by aqueous amidation.

### 2.2. Obtaining Topical Formulations

The preparation of the topical formulation was carried out in three stages as follows: (i) synthesis of nanocapsules functionalized with aptamers and loaded with 5-FU; (ii) obtaining liposomes conjugated with aptamers loaded with 5-FU; (iii) the incorporation of the obtained nanoparticles (nanocapsules, liposomes) in an oil-in-water emulsion.

The nanocapsules were prepared by an interfacial condensation method as described in our previous studies [10]. To obtain them, a synthetic polymer (poly (NVPAI)) and a mixture of two natural polymers (chitosan/aptamer-functionalized chitosan) were used. In our previous studies [10], several types of nanocapsules were obtained and the aptamer-functionalized polymeric nanocapsules, noted as NCA-1, were chosen due to their small diameter (around 133 nm), good release rate of 5-FU, excellent hemocompatibility (hemolysis was around 0.36%, suggesting that the tested formulations did not induce red blood cell lysis) and lack of toxicity.

The liposomes were obtained by a simple method (hydration of the lipid film). To obtain smaller sizes of liposomes, the extrusion method was used after hydration. The liposomes were functionalized on the surface with the AS1411 as already described in our previous study [17]. In the present study, the sample L4Apt-5FU-15, abbreviated as L4, was used for analysis. 

Topical formulation-type cream (noticed as C1) is an oil-in-water emulsion. The NCA-1 or L4 suspensions (in ultrapure water) were added to C1 formulation using a mass ratio of 1/2 (nanoparticles/formulation). To obtain 150 mg of C1–NCA-1 formulation, 100 mg of C1 and 50 mg of NCA-1 nanocapsule suspension loaded with 5-FU (0.34 g 5-FU/1 g nanocapsules) were used [10]. To obtain 150 mg C1–L4 formulation, 100 mg C1 and 50 mg L4 liposome suspension loaded with 5-FU (0.194 g 5-FU/g lipids) were used [37].

### 2.3. Transdermal Diffusion Assays 

**Figure 1:**
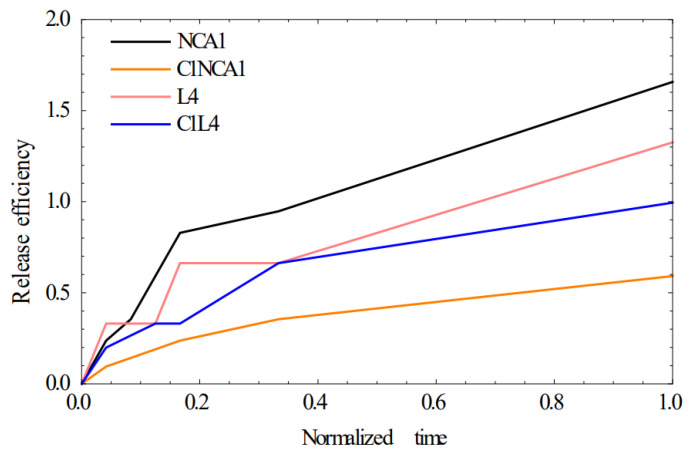
Drug permeation profiles across the Strat-M^®^ artificial membrane .

Kinetic permeation tests were carried out on a vertical Franz diffusion system using a Strat-M artificial membrane [17]. Figure 1 presents the release efficiency from drug-loaded nanoparticles (NCA1 and L4) and drug-loaded nanoparticles mixed into the topical formulations (C1–NCA1 and C1–L4) for 24 h.

The drug permeability (drug released) value after 24 h was 1.66% for NCA-1 and 1.33% for L4. The release efficiency decreased when the drug-loaded functionalized nanoparticles were incorporated into C1 formulations (0.59% for C1–NCA-1 and 0.99% for C1–L4). The release of the drug is influenced both by the type of nanoparticles and by their incorporation into the cream, the cream acting as a new barrier that slows down the drug diffusion.

## 3. Drug Release Mimed as a Multifractal Tunnel Effect

Next, we consider that the delivery system, i.e., nanocapsules or liposomes, is acting as a potential barrier for the drug particles encapsulated within. In general, the scalar potential that describes a potential barrier has the form:

(1a) $$U\left(x\right)=\left\{\begin{array}{c} 0,\;\;\mathrm{if}\;-\infty <x<0\\ U_{0},\;\;\;\mathrm{if}\;0\leq x\leq a\\ 0,\;\;\;\mathrm{if}\;a\leq x\leq +\infty \end{array}\right.\;$$

where $U_{0}$ and a are characteristics of the complex system, i.e., the potential barrier height and its width. When the height $U_{0}$ of the barrier is infinite, the incident drug particles are unable to penetrate it and the particle is “reflected”, while for a finite height, part of the incident drug particles can pass through it.

**Figure 2:**
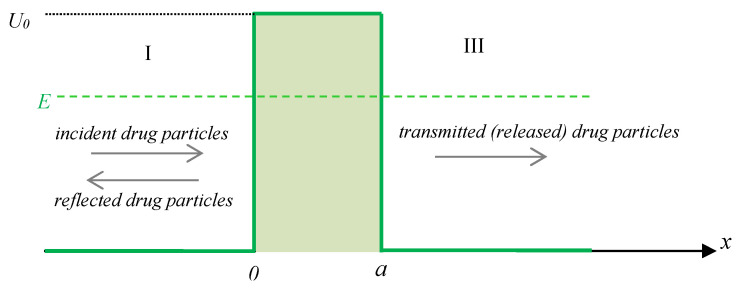
The three physical regions of a potential barrier of height $U_{0}$ and width a (E represents the energy of the incident drug particle).

The potential barrier creates three physical regions with three different behaviors (Figure 2). In the first region, corresponding to −∞<x<0, the incident drug particles move in a potential-free zone and coexist with the reflected particles. In the second region, for 0≤x≤a, part of the incident drug particles that have not been reflected at x=0 pass as transmitted drug particles in a constant potential $U_{0}$ and tunnel through to the third region at x=0, a point from which they move as free particles in a potential-free zone. The number of the incident particles that can tunnel through the barrier depends both on the barrier’s characteristics, i.e., its width a and its height $U_{0}$, and on the energy *E* of the drug particle incident on the barrier [39].

In the following, the tunnel effect is described through Scale Relativity Theory (SRT) [40] as a multifractal tunnel effect. SRT has been applied in describing the drug release from different types of delivery systems, including hydrogels [41,42], magnetic nanoparticles [43,44] and microparticles [45,46]. In this theory, the particles move on continuous but non-differential curves, named fractal curves. Consequently, the physical quantities that characterize the system’s evolution also have fractal character, i.e., they depend both on the spatio-temporal coordinates and resolution scales [47].

In such a context, if we note with $f_{1},f_{2}$ and $f_{3}$ the multifractal functions that describe the three regions of the potential barrier, the following equations can be written:

(1b) $$\frac{\partial^{2}f_{1}}{\partial x^{2}}+k^{2}f_{1}=0,\;\mathrm{for}-\infty <x<0$$

(1c) $$\frac{\partial^{2}f_{2}}{\partial x^{2}}-q^{2}f_{2}=0,\;\mathrm{for}\;0\leq x\leq a$$

(1d) $$\frac{\partial^{2}f_{3}}{\partial x^{2}}+k^{2}f_{3}=0,\;\mathrm{for}\;a\leq x\leq +\infty$$

where

(2) $$k^{2}=\frac{E}{2\lambda^{2}{\left(dt\right)}^{\left[4/g\left(\alpha \right)\right]-2}}\;\mathrm{and}\;q^{2}=\frac{U_{0}-E}{2\lambda^{2}{\left(dt\right)}^{\left[4/g\left(\alpha \right)\right]-2}}$$

are constants that encompass the system’s fractality.

The solutions of the above equations are of the form:

(3a) $$f_{1}\left(x\right)=A_{1}e^{ikx}+B_{1}e^{-ikx}$$

(3b) $$f_{2}\left(x\right)=A_{2}e^{qx}+B_{2}e^{-qx}$$

(3c) $$f_{3}\left(x\right)=A_{3}e^{ikx}$$

where $A_{1},B_{1},A_{2},B_{2},A_{3}$ are arbitrary constants.

The term $e^{ikx}$ corresponds to the multifractal incident states from Region I and to the multifractal emergent states in Region III, while $e^{-ikx}$ corresponds to the multifractal reflected states, which exist only in Region II.

Next, we focus our attention to Region III, corresponding to the existence of the drug particles released from the delivery system.

The multifractal current of the states density in Region I can be written as [48]:

(4) $$J_{1}=2\lambda {\left(dt\right)}^{\left[2/g\left(\alpha \right)\right]-1}k{\left|A_{1}\right|}^{2}$$

while the multifractal current density of the multifractal emergent states density in Region III is:

(5) $$J_{3}=2\lambda {\left(dt\right)}^{\left[2/g\left(\alpha \right)\right]-1}k{\left|A_{3}\right|}^{2}$$

The above results allow the characterization of the tunnel effect, from a fractal point of view, through the multifractal transparency, defined as:

(6) $$T=\frac{J_{3}}{J_{1}}={\left|\frac{A_{3}}{A_{1}}\right|}^{2}$$

The imposition of the coupling conditions in x=0 and x=0, for the functions $f_{i}$ and their derivates, is described as follows:

$$f_{1}\left(0\right)=f_{2}\left(0\right),\;\;\frac{df_{1}}{dx}\left(0\right)=\frac{df_{2}}{dx}\left(0\right),\;f_{2}\left(a\right)=f_{3}\left(a\right),\;\;\frac{df_{2}}{dx}\left(a\right)=\frac{df_{3}}{dx}\left(a\right)\;$$

and leads to the next expression of the multifractal transparency:

(7) $$T-T_{0}=F\cdot \frac{4q^{2}k^{2}}{4q^{2}k^{2}+{\left(q^{2}+k^{2}\right)}^{2}sh^{2}\left(qa\right)}$$

where $T_{0}$ represents an intrinsic transparency of the release environment in relation to the drug considered and F is a multifractal constant, specific to the system, encompassing its complexity and multifractality. With the notations from (2), Equation (7) takes the form:

(8) $$T-T_{0}=F\cdot \frac{4E\left(U_{0}-E\right)}{U_{0}^{2}sh^{2}\left\{{\left[\frac{\left(U_{0}-E\right)}{2\lambda^{2}{\left(dt\right)}^{\left[4/g\left(\alpha \right)\right]-2}}\right]}^{1/2}a\right\}+4E\left(U_{0}-E\right)}$$

To simplify the above equation and its interpretation, it is more convenient to work in a dimensionless coordinate system, i.e.,

(9) $$X_{n}=ka={\left[\frac{E}{2\lambda^{2}{\left(dt\right)}^{\left[4/g\left(\alpha \right)\right]-2}}\right]}^{1/2}a,\;Y_{n}=qa={\left[\frac{\left(U_{0}-E\right)}{2\lambda^{2}{\left(dt\right)}^{\left[4/g\left(\alpha \right)\right]-2}}\right]}^{1/2}a$$

with which the multifractal transparency becomes:

(10) $$T-T_{0}=F\cdot \frac{4X_{n}^{2}Y_{n}^{2}}{4X_{n}^{2}Y_{n}^{2}+{\left(X_{n}^{2}+Y_{n}^{2}\right)}^{2}sh^{2}Y_{n}}$$

By noting the energy ratio $U_{0}/E=m$, the above relation can be written as:

(11) $$T-T_{0}=F\cdot \frac{4\left(m-1\right)}{4\left(m-1\right)+m^{2}sh^{2}X_{n}^{2}\left(m-1\right)}$$

Since the present model implies a field theory with spontaneous symmetry breaking [49,50], the stationary–non-stationary state transition, to which the drug release process can be assimilated, is achieved through an operational procedure as a Wick-type rotation $X_{n}\to \nu_{c}t$, where $\nu_{c}$ is the frequency of interactions within the system and t is the time. In this context, the transparency of the drug delivery system, considered as a potential barrier, becomes:

(12) $$\Delta T=T-T_{0}=F\cdot \frac{4\left(m-1\right)}{4\left(m-1\right)+m^{2}sh^{2}\nu_{c}^{2}t^{2}\left(m-1\right)}$$

**Figure 3:**
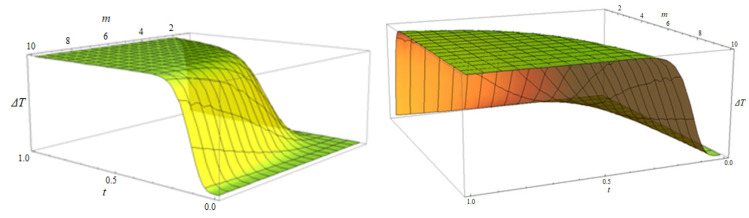
The three-dimensional variation of the multifractal transparency variation ΔT for time t and energy ratio m, from two different perspectives.

The multifractal transparency variation $\Delta T=T-T_{0}$ for time t and energy ratio m is shown in Figure 3.

**Figure 4:**
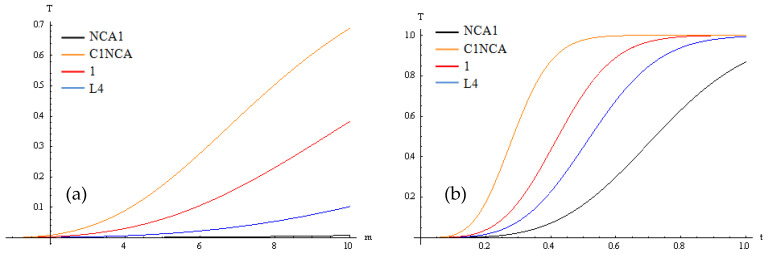
The multifractal transparency variation, in a two-dimensional representation, for energy ratio m (**a**) and time t (**b**) and for t=const. and m=const., respectively.

Considering one variable constant, the multifractal transparency variation for energy ratio m and time t, and for t=const. and m=const. are represented in Figure 4a,b.

The dependence of ΔT on the energy ratio m involves a quasi-linear increase in the multifractal transparency, while its dependence on time t involves an exponential increase.

## 4. Model Validation

By fitting the theoretical model with the experimental data, in terms of release efficiency, specific information on the drug delivery systems at a microscopic level, such as intrinsic transparences $T_{0}$ to the drug molecules, multifractal constants F as indicators of the system’s complexity, the frequency of interactions $\nu_{c}$ within the system and the energy ratio m between potential barrier energy and the energy of drug molecules, were obtained (Table 1).

A comparative analysis of the obtained values shows that both types of nanoparticles have the same intrinsic transparence to the 5-FU drug molecules, while when immersed in formulations, it decreases; this is somewhat explained, since the emulsions induce a smaller “transparency” of the release system.

A similar decrease is observed also for the multifractal constant; this actually reflects the functional multifractality of the system, the processes being slower in formulations.

The energy ratio between potential barrier energy and the energy of drug molecules also decreases and its effect is observed in the drug release rate, which is lower in the formulations case.

The only system characteristic that increases in formulations is the frequency of interactions in systems, an anticipated result, considering the increase in the number of system particles.

When comparing the two types of nanoparticles, nanocapsules vs. liposomes, one can see that they have similar intrinsic transparence and multifractality. Significant differences appear for the frequency of interactions and energy ratio, which are smaller for liposomes, revealing that they are systems with less energy embedded within them, most likely as a consequence of the functional equilibrium following the self-organization of the liposome as a lipid bilayer.

**Figure 5:**
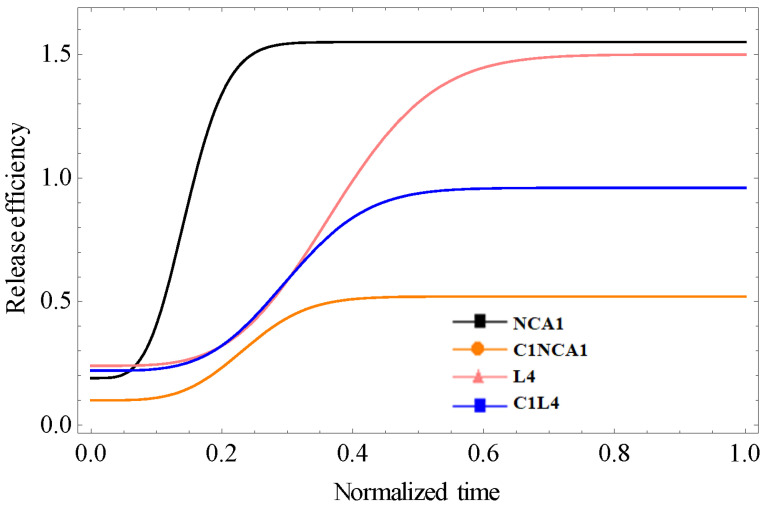
Comparative plots of experimental data and theoretical curves.

The comparative plots of experimental data and their theoretical model fit are illustrated in Figure 5.

## 5. Discussion of Differentiability to Nondifferentiability in Drug Release Kinetics

The previous results can lead to possible new theoretical routes for describing drug release dynamics. Indeed, mathematically, it is easy to design a pharmaceutical system that can be used to induce the effect of device design parameters on drug release kinetics. By using mathematical models, a quantitative analysis of drug release rate data can be obtained. The selection of a particular model is dictated by the predictive ability and accuracy of the model. Drug release is the process by which a drug becomes a product subject to absorption, distribution, metabolism and excretion (ADME). Drug release is described in several ways:

- Immediate-release drug products allow drugs to dissolve without delay or prolonging drug dissolution or absorption;
- Extended-release drug products that are included in the modified-release dosage form. Delayed release is the release of the drug at a time other than immediately after administration. Extended-release products are designed to make the drug available for a longer period of time after administration;
- Controlled release includes extended- and pulsatile-release products. This mechanism involves the release of well-defined amounts of the drug at a specified time at various precise intervals.

In vitro dissolution studies are an important step in the development of new drugs. Several theories and models describe the drug release profile from a perspective of pharmaceutical dosage [7,8,10].

Determinism does not necessarily imply regular (periodic motions, self-structures, etc.) or predictable behavior in polymeric systems [14,15]. In linear analysis, on which polymer physics relies almost exclusively, unbounded predictability has been an automatic quality of polymer system dynamics. The development of nonlinear analysis and the discovery of the laws governing chaos have shown that the reductive analysis method, which is applicable to polymer physics, has been limited so far, but its applicability has also been limited. Unlimited predictability is not an attribute of polymer systems but is, in fact, a natural consequence of their simplification by the linear approach. There are only a few behaviors wherein nonlinearity and chaos highlight common manifestations (superstructure, flammability, etc.), namely a universality in the laws dictating the dynamics of polymeric systems [14,15].

The behavior of polymers is determined by the conditions under which they are produced and the conditions under which they are used [10,11,12]. The nonlinearity and chaoticity of any polymer system are both structural and functional, and are determined by the interactions between its microscopic–macroscopic, local–global and individual–collective structural units. In such a framework situation, the universality of the dynamic laws of polymeric systems becomes natural and must be reflected in the mathematical procedures used [42,46]. There are authors who increasingly discuss the holographic implementation involved in the description of polymer dynamics (fractal paradigm of nature) [40].

The usual physical models used in describing the dynamics of polymeric systems are based on an otherwise unfounded assumption of the differentiability of the physical quantities used to describe their evolution [39,40]. The success of these models must be understood sequentially, in areas where differentiability and integrability are still valid [6] Differentiable and integrable mathematical procedures fail when we intend to describe the dynamics of physical systems involving only nonlinearity and chaoticity. In order to describe the dynamics of these physical systems, it is necessary to explicitly introduce scale resolution into the expressions of the physical variables and implicitly into the expressions of the fundamental equations governing these dynamics [39,40].

This means that any physical variable, dependent—by usual mathematical procedures—on both spatial and time coordinates, also depends on a scale resolution. In other words, for example, instead of working with a single physical variable, described by a strictly undifferentiable mathematical function, we work only with approximations of this mathematical function obtained by averaging it at different scale resolutions. Consequently, any physical variable used to describe the dynamics of polymeric systems functions as the limit of a family of mathematical functions, the function being undifferentiable for zero scale resolution and differentiable for nonzero scale resolution [39,40].

This way of describing the dynamics of polymeric systems, for which measurements are performed at finite scale resolutions, obviously implies the development of both new geometric structures and physical theories (consistent with these geometric structures) for which the laws of motion, invariant to time coordinate transformations, are also invariant to transformations with respect to scale resolution [40]. In our opinion, such a geometric structure is the one based on the concept of fractals and the corresponding physical model described in the Scale Relativity Theory [40]. From this perspective, the holographic implementation in the description of the dynamics of polymer systems are made explicit based on the description of the dynamics of the structural units of any polymer by continuous but undifferentiable curves (fractal curves) [39,40].

Some consequences are obvious:

- Motions with “ties” (with constraints) on continuous and differentiable curves in a Euclidean space are substituted by motions free of any constraints on continuous but non-differentiable curves in a fractal space;
- Motion curves act both as geodesics of a fractal space and as streamlines of a fractal fluid;
- The structural units of any polymeric system are substituted with their own geodesics, with any external constraint being interpreted as a selection of geodesics on the basis of local–global/whole–part compatibility, etc.;
- For large time scales, relative to the inverse of the largest value of the Lyapunov exponent [47,49], deterministic trajectories can be substituted by families of potential trajectories so that the concept of position is replaced by that of probability density [47,49];
- Taking into account that the descriptions of drug release dynamics are given by continuous and differentiable curves (fractal/multifractal curves) and that these curves have the self-similarity property (one part reflects the whole and vice versa), then “holographic” descriptions of the drug release dynamics become functional.

## 6. Conclusions

The drug release from nanoparticles (polymeric nanocapsules and liposomes) functionalized on the surface with an aptamer, included also in cream-type formulations, was studied experimentally and theoretically. The efficiency of drug release is affected both by the type of nanoparticles (the nanocapsules being more efficient in releasing the drug) and by their incorporation into the cream, the cream acting as a new barrier that slows down drug diffusion.

The theoretical model, based on the multifractal tunnel effect in the Scale Relativity Theory, allowed us to analyze the system’s behavior at a microscopic level and to evaluate microscopic characteristics of the system, such as intrinsic transparences to the drug molecules, multifractal constants as indicators of the system’s complexity, the frequency of interactions within the system and the energy ratio between potential barrier energy and the energy of drug molecules. The formulations proved to have smaller “transparency” for the drug molecules.

When comparing the two types of nanoparticles, nanocapsules vs. liposomes, similar intrinsic transparence and multifractality were found. Significant differences appear for the frequency of interactions and energy ratio, which are smaller for liposomes, revealing that they have less energy embedded within, most likely as a consequence of the functional equilibrium following the self-organization of the liposome as a lipid bilayer.

## Figures and Tables

**Table 1:** The specific microscopic characteristics of the drug delivery systems.

Sample Code	$T_{0}$	F	$\nu_{c}$	m
**NCA1**	1.52	1.36	3.22	4.44
**C1NCA1**	0.52	0.42	4.34	1.84
**L4**	1.52	1.26	1.80	2.90
**C1L4**	0.96	0.74	3.12	2.00

## Data Availability

The data that support the findings of this study are available on request from the corresponding author.
